# Brain ventricle morphology markers in predicting shunt surgery outcome in idiopathic normal-pressure hydrocephalus

**DOI:** 10.1186/s12987-026-00788-4

**Published:** 2026-04-09

**Authors:** Andrius Penkauskas, Eemeli Kuukkanen, Anssi Lipponen, Juhana Hakumäki, Juha Koikkalainen, Jyrki Lotjonen, Lauri Erkkilä, Jussi Tohka, Pauli Miettinen, Ville Leinonen

**Affiliations:** 1 School of Computing, University of Eastern Finland, Kuopio, 70211 Finland; 2 Faculty of Health Sciences, University of Eastern Finland, Kuopio, 70211 Finland; 3 Institute of Biomedicine, University of Eastern Finland, Kuopio, 70211 Finland; 4 Institute of Clinical Medicine–Radiology, University of Eastern Finland, Kuopio, 70211 Finland; 5 Combinostics Ltd., Tampere, 33100 Finland; 6 A.I. Virtanen Institute for Molecular Sciences, University of Eastern Finland, Kuopio, 70211 Finland

## Abstract

**Background:**

Idiopathic normal pressure hydrocephalus (iNPH) is characterized by a clinical triad of symptoms: abnormal gait, memory problems, and urinary incontinence. Neuroimaging plays a crucial role in diagnosing iNPH. However, current radiological markers, though indicative, are not definitive, suggesting the limited capacity of these indices to capture mechanisms associated with iNPH and the reversibility of the symptoms.

**Aims:**

This study aims to (1) determine the geometric features of the lateral ventricles, (2) develop a quantitative method for three-dimensional analysis, and (3) test the ability to predict response to shunt surgery. By examining these features as potential diagnostic markers, this research seeks to enhance the understanding of morphometric characteristics in iNPH, thereby paving the way for improved patient selection for surgical intervention.

**Methods:**

Our study contained 170 patients (95 shunt responders and 75 non-responders) from the Kuopio NPH registry. Our inclusion criteria required pre-surgery and one-year post-surgery symptom assessments alongside preoperative anatomical magnetic resonance imaging (MRI). Volumetric brain segmentations were performed using cNeuro software on T1-MRI images, followed by the generation of 3D lateral ventricle meshes for geometric feature extraction. The classification task employed the LogitNet machine learning model to analyze 27 geometric features. Model performance evaluation utilized repeated nested cross-validation (10 rounds) with five inner folds for parameter tuning and five outer folds for model evaluation. Additionally, we generated a ranking of feature importance based on the LogitNet L1 regularization coefficients.

**Results:**

Our analysis revealed that LogitNet achieved an AUC of 0.661 (SD = 0.066) performance across 10 rounds of cross-validation in predicting the shunt surgery response. The most prominent feature contributing to the model’s prediction was asphericity.

**Conclusion:**

Our analysis suggests that the proposed set of features, especially asphericity, effectively captures valuable information linked to the reversibility of iNPH.

## Introduction

Idiopathic Normal Pressure Hydrocephalus (iNPH), also called Hakim’s disease, is a progressive but potentially treatable brain condition that usually affects the elderly, with its etiology and pathogenetic mechanisms yet to be determined Relkin et al. [34]. Early detection of iNPH is critical since surgical interventions can improve or even reverse disease symptoms Adams et al. [1]; Relkin et al. [34]; Nakajima et al. [29]; Andrén et al. [3]. Commonly, it is characterized by a clinical triad of symptoms: abnormal gait, deterioration in cognition (dementia), and urinary urgency or incontinence Relkin et al. [34]; Nakajima et al. [29].

Even if clinical symptoms are present, neuroimaging is mandatory for diagnosing iNPH, as it provides critical information for accurate disease classification Nakajima et al. [29]. Both computed tomography (CT) and magnetic resonance imaging (MRI) modalities can be used to visualize anatomical changes in the brain associated with iNPH. However, the MRI technique is preferred Relkin et al. [34].

Traditionally, two-dimensional (2D) neuroimaging markers such as the Evans Index (EI), callosal angle (CA), and brain-to-ventricle ratios (BVR) are important tools in the diagnostic process for iNPH, although they are not definitive predictors Relkin et al. [34]; Grahnke et al. [15]; Agerskov et al. [2]; Kockum et al. [19]; Nakajima et al. [29]; Thavarajasingam et al. [37]; Virhammar et al. [40]; Kojoukhova et al. [21]. Although the combination of CA and EI effectively distinguishes patients with iNPH from controls Miskin et al. [27].

A more complex marker, disproportionately enlarged subarachnoid-space hydrocephalus (DESH) Hashimoto et al. [16], is highlighted in the third edition of Japanese guidelines for iNPH management as a crucial imaging feature for diagnosing iNPH Nakajima et al. [29], with potential prognostic value for predicting neurological improvement and overall prognosis following surgery Shinoda et al. [35]. Still, the effectiveness of DESH as a predictor of shunt response is debated Agerskov et al. [2]; Thavarajasingam et al. [37]. Additionally, both the DESH-positive and DESH-negative populations were responsive to shunting, raising doubts about the reliability of DESH as a standalone predictive marker Craven et al. [4]; Agerskov et al. [2]. Moreover, the subjective nature of DESH evaluation lacks consistency; however, if validated through further research, this issue may be resolved with the recently proposed quantitative assessment method Yamada et al. [45]. Furthermore, a recent study indicates that the combination of CA, DESH, and the tap test enhances the prediction of shunt surgery in iNPH patients Gao et al. [10].

The review of radiological predictors for shunt responses in iNPH highlights the emergence of automated deep learning and machine learning techniques that utilize brain image segmentation and three-dimensional (3D) morphometric or volumetric analysis. These methods showed promising yet inconsistent results Thavarajasingam et al. [37]. Volumetric brain segmentations can be transformed into smooth 3D surface meshes that outline the boundaries of the structural segmentation Levine et al. [23]. Analyzing these surface meshes is becoming increasingly important in the field of neurodegenerative diseases, such as Alzheimer’s, Huntington’s, and Parkinson’s disease, as it allows researchers to examine subtle details across different brain regions Martí-Juan et al. [25]; Miller et al. [26]; Faria et al. [7]; Nemmi et al. [30]. However, there is a lack of emphasis on 3D mesh analysis in the iNPH domain. Despite diagnostic advances, predicting shunt responsiveness in iNPH remains challenging due to the condition’s complexity and the limitations of current diagnostic markers Agerskov et al. [2]; Kockum et al. [19]; Nakajima et al. [29]; Thavarajasingam et al. [37]. Current radiological markers, though indicative, do not fully capture the mechanisms associated with the reversibility of the iNPH Agerskov et al. [2].

Therefore, the current study proposes a novel set of radiological markers for quantifying the shape of the lateral ventricle. In designing this set, we aimed to identify the geometric characteristics of the lateral ventricles, develop a quantitative method for 3D mesh analysis, and evaluate the ability of these features to predict responses to shunt surgery.

To achieve this, we developed an automated feature extraction pipeline that allowed us to measure the curvature and both local and global characteristics of the lateral ventricles in patients diagnosed with iNPH. The extracted features were subsequently employed to classify responses to shunt surgery.

## Methods

### iNPH cohort

The patient population in the iNPH cohort was gathered from the catchment area of Kuopio University Hospital (KUH). This area has a total population of 800,000 people and geographically includes Central Finland, Northern Karelia, and the Northern, Southern, and Eastern Savonia regions. This study was accepted by the institutional research ethics committee of the KUH and performed according to the 1964 Helsinki declaration and its later amendments or comparable ethical standards. All participants provided written informed consent.

Patients were referred to the neurosurgery department at KUH based on diagnoses made by neurologists or geriatricians. To qualify for referral, patients needed to exhibit at least one of the three primary symptoms: gait disturbance, impaired cognition, or urinary incontinence. The main imaging modality utilized was MRI, which revealed enlarged brain ventricles and a decreased callosal angle as supportive markers for the diagnosis. Additionally, it was necessary to rule out any other more apparent etiology for the symptoms.

Diagnoses were made following the KUH iNPH protocol Junkkari et al. [17]. The three-step prognostic part of the KUH iNPH protocol has been in use since 2010. The Lumbar tap test (LTT) is the first-line diagnostic test in the protocol, as it is performed for every patient suspected of iNPH. The CSF removal by LTT acts analogously to a shunt. At least 20% improvement in the repeated 10-meter gait speed test is considered positive and favours shunt surgery. In the case of a negative LTT, an infusion test is the second step in the protocol. Outflow resistance is measured by infusing Ringer’s solution, which acts as artificial cerebrospinal fluid (CSF) via lumbar puncture into the subarachnoid space. A pressure difference equal to or higher than 12 mmHg/(ml/min) compared to baseline intracranial pressure (ICP) indicates an iNPH diagnosis. In the case of negative LTT and infusion tests, a clinical re-evaluation is considered.

**Fig. 1:**
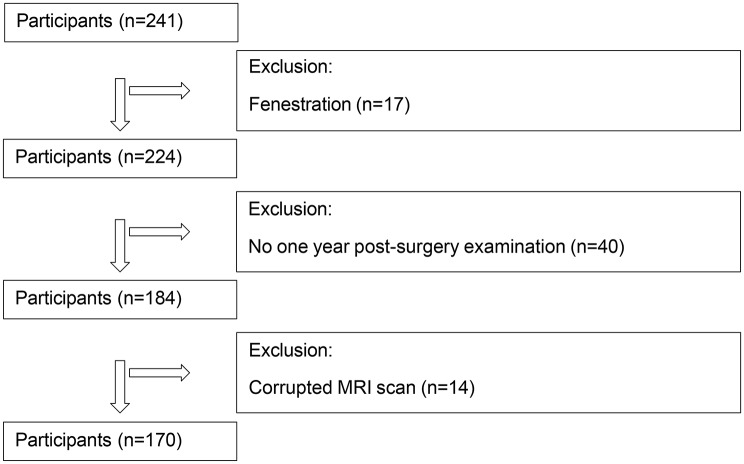
Participant selection and exclusion criteria flowchart

The final study cohort consisted of 170 patients who underwent shunt surgeries between January 2013 and November 2022. This group included 95 shunt responders and 75 non-responders, selected from a larger cohort based on specific inclusion and exclusion criteria. The inclusion criteria mandated that patients had undergone shunt surgery and received a preoperative and one-year postoperative assessment, along with a preoperative brain MRI scan; conversely, exclusion criteria encompassed cases involving NPH with a potentially known origin (sNPH) or fenestration surgery related to longstanding overt ventriculomegaly in adults (LOVA) Palandri et al. [31], as well as instances of low-quality MRI scans or subpar-quality lateral ventricle segmentations. The flowchart of selection and exclusion criteria is presented in Fig. 1.

In contrast to curated datasets, this study utilized real-world data. T1-weighted MRI scans were obtained from multiple locations using different MRI scanners of various manufacturers. To ensure consistent image quality, the MRI images were assessed manually. Non-3D T1w scans, partial brain scans, and those exhibiting significant noise or motion blur that rendered them unsuitable for segmentation were excluded from the analysis.

The average age of the subcohort population was 73.7 years (SD = 6.71). Within this population, 54.7% were female, and 45.3% were male. Biopsies were obtained with shunt surgeries, during which samples were collected from cortical tissue before inserting the CSF catheter. A neuropathologist subsequently graded these samples for amyloid-beta (A*β*) and hyperphosphorylated tau (HP*τ*) immunoreactivity Junkkari et al. [17]. The presence of A*β* and HP*τ* pathologies, and clinical comorbidities is summarized in Table 1.

**Table 1:** Subcohort conditions

Condition	Count	Percentage
Hypertension	114	67.1
Diabetes	68	40.5
Presence of only A*β*	68	40.7
Presence of only HP*τ*	8	4.79
Presence of A*β* and HP*τ*	18	10.8

Table 2 outlines the radiological characteristics of iNPH based on measures from the Radscale Kockum et al. [18]. It summarizes key signs of iNPH, with scores stratified by positive and negative responses, thereby illustrating the profiles of responders and non-responders.

**Table 2:** Radiological features stratified by shunt response

Measure	Category	Negative Response	Positive Response
Evans index		0.38 (SD = 0.04)	0.38 (SD = 0.04)
Narrow sulci	Normal	25 (14.7%)	26 (15.3%)
	Parafalcine	3 (1.8%)	8 (4.7%)
	Vertex	47 (27.6%)	61 (35.9%)
Sylvian fissures	Normal	9 (5.3%)	11 (6.5%)
	Enlarged	66 (38.8%)	84 (49.4%)
Focally enlarged sulci	Not present	33 (19.4%)	46 (27.1%)
	Present	42 (24.7%)	49 (28.8%)
Temporal horn width (left)		8.26 (SD = 4.05)	7.8 (SD = 2.79)
Temporal horn width(right)		8.23 (SD = 2.84)	7.65 (SD = 2.66)
Callosal angle		59.12 (SD = 16.27)	54.39 (SD = 14.79)
Periventricular hypodensities	Not present	11 (6.5%)	7 (4.1%)
	Frontal horn caps	18 (10.6%)	35 (20.6%)
	Confluent areas	46 (27.1%)	53 (31.2%)
Radscale total		9.48 (SD = 1.66)	9.63 (SD = 1.75)

The Evans index, temporal horn width, callosal angle, and Radscale total are presented in absolute values with standard deviation. The narrow sulci, Sylvian fissures, enlarged sulci, and periventricular hypodensities are presented in counts and categorized according to the Radscale

### Assessment

A modified Finnish version of the 12-point iNPH grading scale (iNPHGS) was utilized to evaluate the severity of symptoms Kubo et al. [22]. This clinician-rated scale allows for a detailed assessment of the three main symptoms based on interviews with patients or their caregivers and observations by the physician. A high score on the iNPHGS indicates a greater expression of the clinical triad of symptoms. A reduction of at least one point in the iNPHGS is considered a clinically significant improvement in the patient’s condition. This scale was used to encode a surgical outcome as binary for further analysis.

In addition to iNPHGS, cognitive impairment was evaluated utilizing the Mini-Mental State Examination (MMSE) Folstein et al. [8]. The MMSE provides a maximum score of 30, which reflects normal cognitive performance. Unfortunately, not all patients underwent MMSE. For a gait speed test, an improvement of at least 20% in gait velocity was considered a favorable outcome Table 3.

**Table 3:** Preoperative and 12-month follow-up assessment

Metric	Preoperative assessment		12-month postoperative assessment	
	Mean	SD	Mean	SD
iNPHGS	5.96 (*n* = 170)	2.65	5.05 (*n* = 170)	3.04
MMSE	23.6 (*n* = 165)	4.01	24.6 (*n* = 148)	4.05
Gait speed	2.20 (*n* = 170)	0.99	1.68 (*n* = 170)	1.20

### Proposed framework

**Fig. 2:**
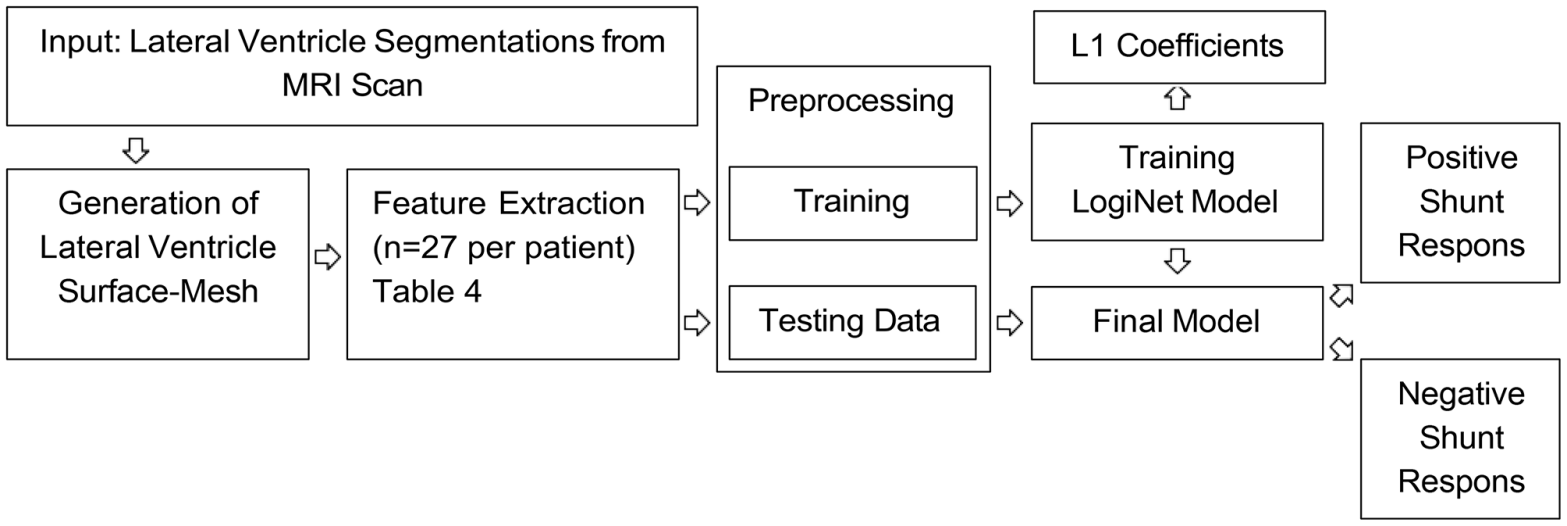
Flowchart of the proposed framework to predict the outcomes of iNPH shunt surgery based on features derived from the lateral ventricles

We propose a five-step protocol to predict outcomes of iNPH shunt surgery based on features derived from the lateral ventricles. This framework consists of the following steps: generation of the lateral ventricle 3D meshes, extraction of mesh features, data preprocessing, classification, and ranking of feature importance. The details of the proposed framework are illustrated in Fig. 2 .

In our analysis, regional lateral ventricle volumes were determined using a fully automated multi-atlas segmentation tool, cNeuro (Combinostics Ltd, Tampere, Finland), from T1 MRI images Koikkalainen et al. [20]; Lötjönen et al. [24]. This tool segments T1 images into 133 brain regions based on 79 manually segmented atlases (Neuromorphometrics Inc., Massachusetts, USA).

#### 3D mesh generation

We used Vedo Musy et al. [28], a Python module for scientific analysis of 3D objects and point clouds, that utilizes a 3D version of the Surface Nets algorithm Gibson [11] to create an isocontour surface from lateral ventricle segmentations, resulting in a triangulated mesh. Given that our dataset comprised MRI scans acquired from various scanners with differing resolutions, we standardized the granularity of all meshes by decimating (downsampling) the number of vertices in each mesh to the target value of 24,000. In a small number of cases, where the total count of vertices was below 24,000, the decimation process was preceded by mesh subdivision, dividing each triangle into four triangles and approximating their smoothed surface utilizing the Trimesh Python module Dawson-Haggerty [5]. Additionally, we employed improved Laplacian smoothing Desbrun et al. [6]; Vollmer et al. [42] with five iterations and a lambda parameter set to 1.0, utilizing the Trimesh library Dawson-Haggerty [5] to reduce noise.

#### Feature extraction

**Fig. 3:**
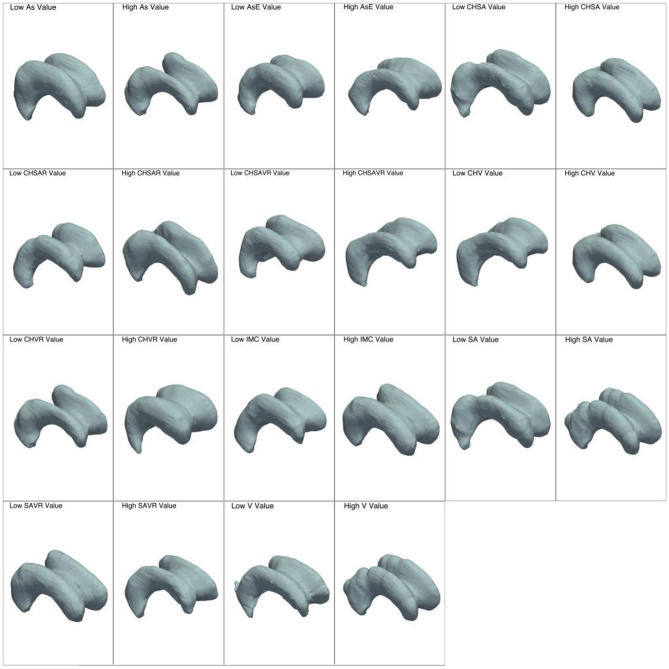
Characterization of each global feature with two distinct meshes: the first corresponds to the 10th quantile of the feature values, reflecting lower values, while the second corresponds to the 90th quantile of the feature values, indicating higher values

**Fig. 4:**
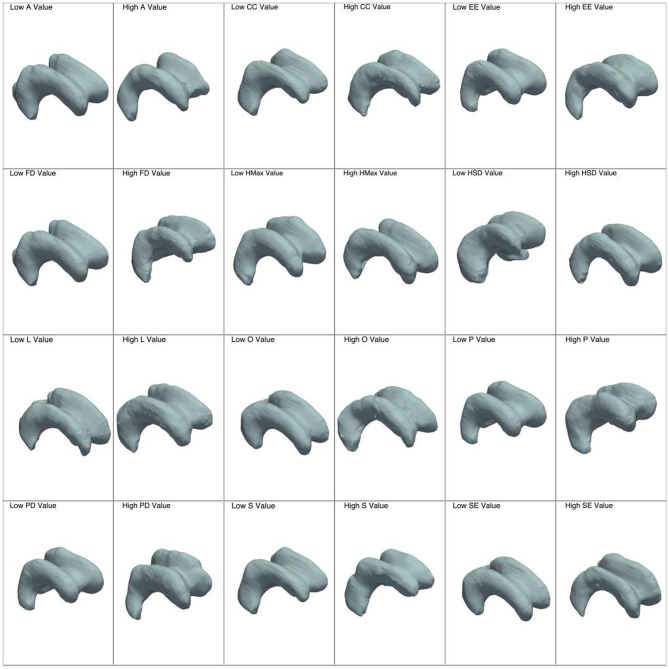
Characterization of each local feature with two distinct meshes: the first corresponds to the 10th quantile of the feature values, reflecting lower values, while the second corresponds to the 90th quantile of the feature values, indicating higher values

**Fig. 5:**
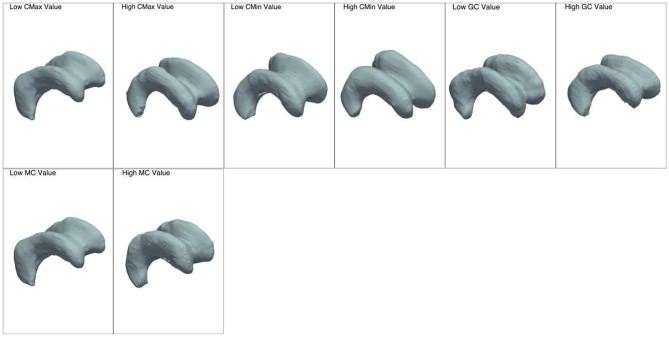
Characterization of each curvature feature with two distinct meshes: the first corresponds to the 10th quantile of the feature values, reflecting lower values, while the second corresponds to the 90th quantile of the feature values, indicating higher values

We identified and extracted 27 features from each participant’s lateral ventricles, which were subsequently categorized into three distinct groups based on their inherent characteristics. These groups include global features, which capture the overall form of the 3D object; local features, which focus on specific localized aspects of shape; and curvature features, which assess variations in curvature. A summary of these features is presented in Table 4; Figs. 3, 4 and 5 .

##### Global features

We computed a set of global shape features that capture the overall properties of the object using the entire mesh. Our global feature set comprises eleven features: point cloud asphericity (As) and asphericity error (AsE) Musy et al. [28], integral mean curvature (IMC) Dawson-Haggerty [5], surface area (SA) and mesh volume (V) from the PyVista module Sullivan and Kaszynski [36], surface area to volume ratio (SAVR), convex-hull surface area (CHSA), convex-hull surface area to ventricle surface area ratio (CHSAR), convex-hull volume (CHV), convex-hull volume to ventricle volume ratio (CHVR), and convex-hull surface area to volume ratio (CHSAVR). The ventricles’ convex-hull was computed utilizing the Trimesh library Dawson-Haggerty [5].

**Table 4:** List of features derived from the lateral ventricles

Global Features	Local Features	Curvature Features
Asphericity (As)	Anisotropy (A)	Gaussian Curvature (GC)
Asphericity Error (AsE)	Change of Curvature (CC)	Maximum Curvature (CMax)
Convex-Hull Surface Area (CHSA)	Eigen-entropy (EE)	Mean Curvature (MC)
Convex-Hull Surface Area to Ventricle Surface Area Ratio (CHSAR)	Height Standard Deviation (HSD)	Minimum Curvature (CMin)
Convex-Hull Surface Area to Volume Ratio (CHSAVR)	Farthest Distance (FD)	
Convex-Hull Volume (CHV)	Linearity (L)	
Convex-Hull Volume to Ventricle Volume Ratio (CHVR)	Maximum Height (HMax)	
Integral Mean Curvature (IMC)	Omnivarience (O)	
Surface Area (SA)	Planarity (P)	
Surface Area to Volume Ratio (SAVR)	Point Density (PD)	
Volume (V)	Sphericity (S)	
	Sum of eigenvalues (SE)	

##### Local features

We computed a set of pointwise local 3D shape features to effectively capture the spatial arrangement and relationships among neighbouring points. To achieve this, we constructed KDTrees from the point clouds and employed the search_radius_vector_3d function from the Open3D Python module Zhou et al. [46]. Following the computation of the 24000 values, we aggregated these results by calculating their average, yielding a single scalar value. Our local feature set is based on the work of Weinmann et al. [43]. Eight features—linearity (L), planarity (P), sphericity (S), omnivariance (O), anisotropy (A), eigen-entropy (EE), sum of eigenvalues (SE), and change of curvature (CC) West et al. [44]; Pauly et al. [32] —are derived from the eigenvalues of the 3D structure tensor calculated at each point i and its neighbours. The remaining four features—the farthest distance (FD), local point density (PD), maximum height difference (HMax), and height standard deviation (HSD)—are based on geometric properties as described by Weinmann et al. [43]. The code to compute these features is sourced from the Extract Features from 3D Meshes for Machine Learning repository GitHub—IvanNik17 [13].

##### Curvature features

We computed a set of pointwise curvature features to describe the spatial variation in surface roughness, which were then averaged across the surface to obtain a single scalar value. This feature set included four curvature measures: Gaussian curvature (GC), mean curvature (MC), maximum curvature (CMax), and minimum curvature (CMin), all derived using the PyVista module Sullivan and Kaszynski [36] .

#### Preprocessing

We applied a QuantileTransformer from the SciKit-Learn library Pedregosa et al. [33] to transform feature distributions, effectively reducing the influence of outliers and rendering variables measured at different scales comparable.

#### Classification & feature importance

For the joint classification and feature selection task, we used logistic regression with LASSO penalty (LogitNet) Friedman et al. [9], employing the Python GLM-NET library GitHub-civisanalytics [12]. LogitNet couples logistic regression with an L1 penalty, automatically forcing the coefficients of redundant or irrelevant features to zero. This process leads to joint feature selection and parameter estimation for the logistic regression model. It is performed for various values of the regularization parameter (*λ*), with the optimal value of *λ* being determined using nested cross-validation Friedman et al. [9]. For evaluation, we implemented a 10-times repeated nested cross-validation (CV) framework with five inner and five outer folds to ensure robust and generalizable results Tohka and Van Gils [39]. The inner CV loop optimized LogitNet parameter *λ* using ROC-AUC as the scoring criterion, while the outer loop evaluated performance using a participant split of 136 for training and 34 for testing in each fold.

We evaluated the importance of the features based on the selection frequency within repeated cross-validation. While there are several possibilities to evaluate the importance of the features of the regularized linear classifiers Gómez-Verdejo et al. [14], such as the standardized coefficient weights, we posit that the selection frequency provides a simple and interpretable feature importance measure when coupled with the LogitNet model.

## Results

### Feature characterization via quantile-based visualization

Features extracted from 3D mesh representations of the lateral ventricles were visualized by examining their values at specific quantile levels (10th and 90th quantiles) and grouped according to their category.

Figure 3 illustrates a set of global features providing visual cues regarding lateral ventricle differences when feature values approach opposite extreme values.

Figure 4 presents a collection of visualizations of the lateral ventricles related to the average values of local features approaching the opposite ends.

Figure 5 demonstrates a series of visualizations of the lateral ventricles associated with the average values of curvature features approaching the contrasting ends.

### Feature distribution by response variable

**Fig. 6:**
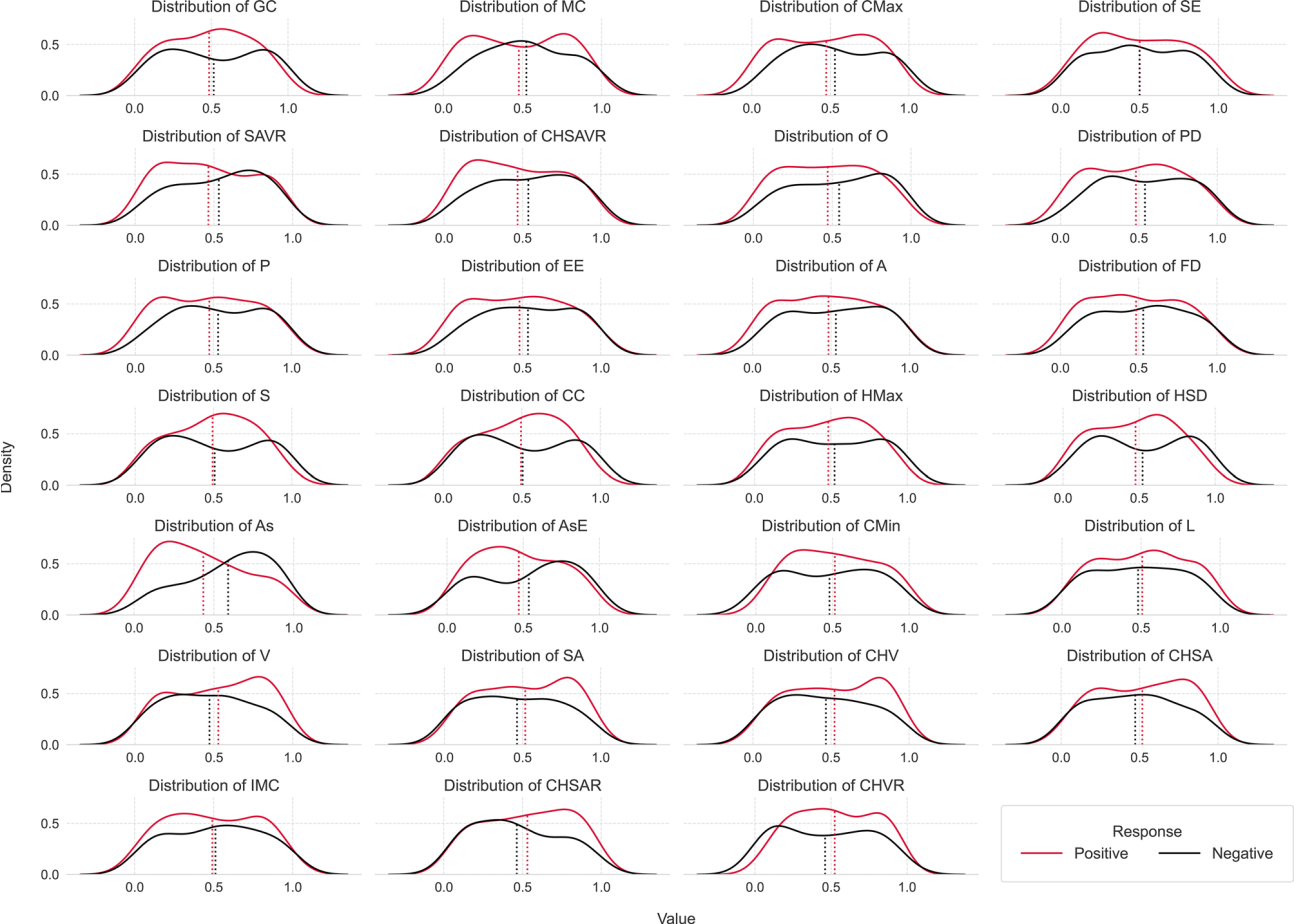
Kernel Density Estimation plots for each feature, stratified by response variable

Figure 6 presents Kernel Density Estimation (KDE) plots for each feature, stratified according to surgical outcomes—specifically, positive and negative responses. These plots serve as a valuable visual tool for contrasting the differences in value distributions between responders and non-responders across each feature. Most of the KDE plots revealed heavy tails on the opposite ends, depending on the response. However, their mean values remained close.

### Feature correlation and hierarchical clustering

**Fig. 7:**
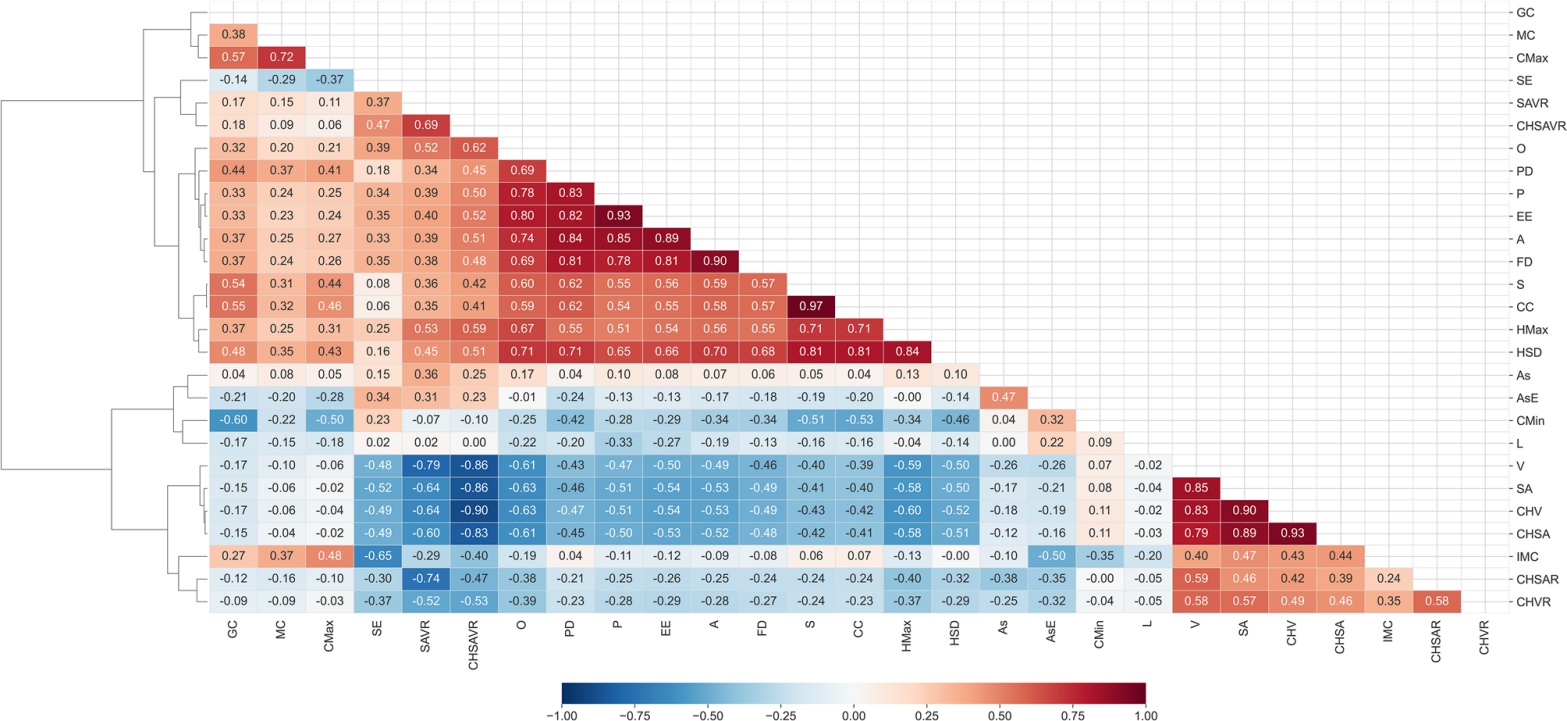
Kendall’s rank correlation heat map with hierarchical clustering dendrogram

Kendall’s rank correlation was used to capture the monotonic relationships between features. These correlations are visualized in Fig. 7. Features were then hierarchically clustered using Ward’s method from the SciPy module Virtanen et al. [41], with the resulting dendrogram highlighting their interrelationships. The features in the figure are sorted based on this clustering, offering a structured view of their correlations. Clustered correlations in Fig. 7 indicate two larger positive correlation clusters and one negative cluster.

### Classification performance & feature importance

Metrics of ROC-AUC, balanced accuracy, sensitivity, and specificity were computed, with their mean and standard deviation (SD) presented in Table 5. All metrics used in the evaluation demonstrated performance that considerably exceeded chance levels.

**Table 5:** Experiment results. The mean and SD of ROC-AUC, balanced accuracy, sensitivity, and specificity from repeated cross-validation

Metric	Mean	SD
ROC-AUC	0.661	0.066
Balanced Accuracy	0.638	0.053
Sensitivity	0.627	0.084
Specificity	0.649	0.095

The mean and SD of ROC-AUC, balanced accuracy, sensitivity, and specificity from repeated cross-validation

**Fig. 8:**
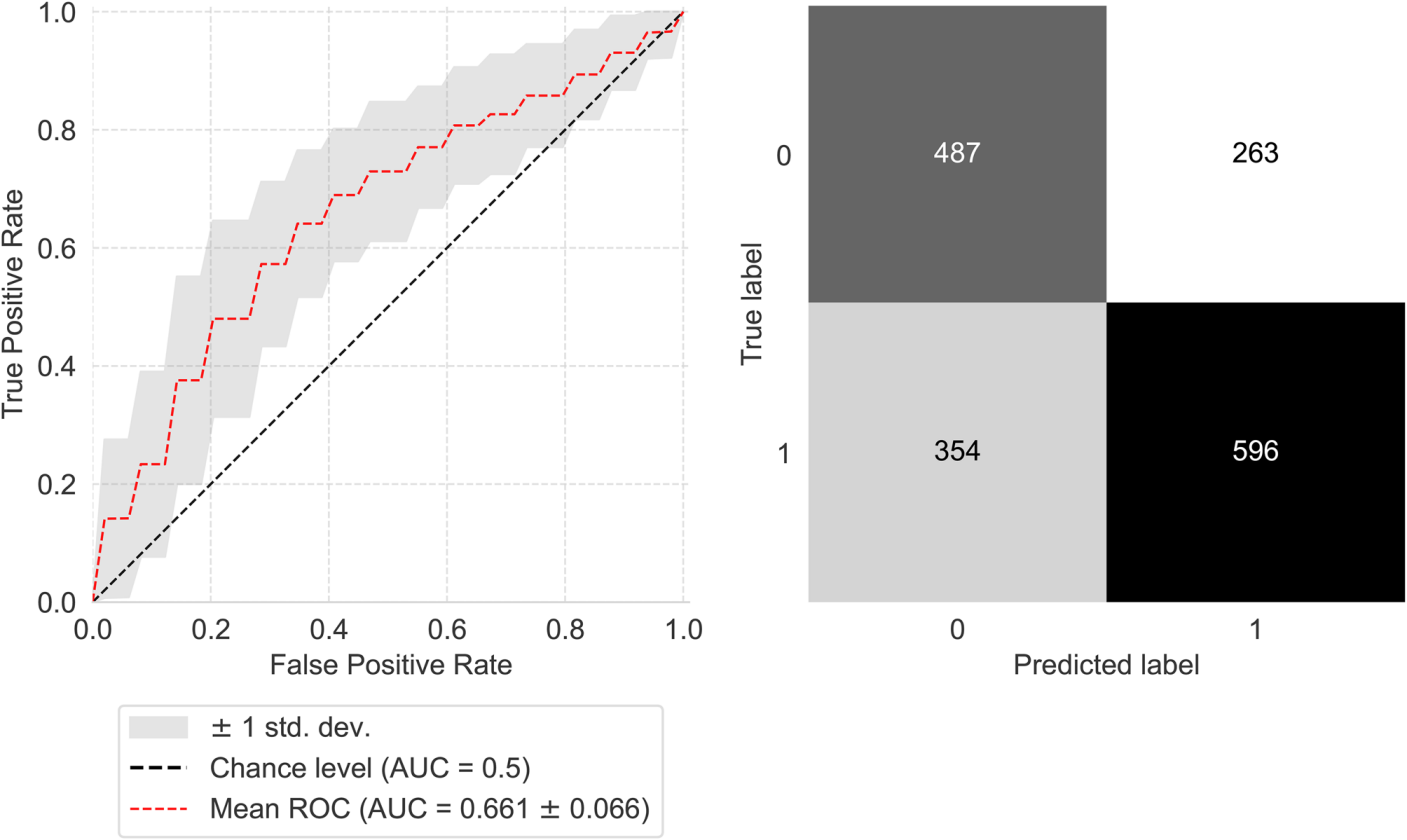
Plot of ROC-AUC values showing the average and standard deviation across 50 folds, along with the confusion matrix

Furthermore, Fig. 8 demonstrates the mean ROC-AUC along with the SD to represent the model’s discriminative ability, as well as the confusion matrix.

**Fig. 9:**
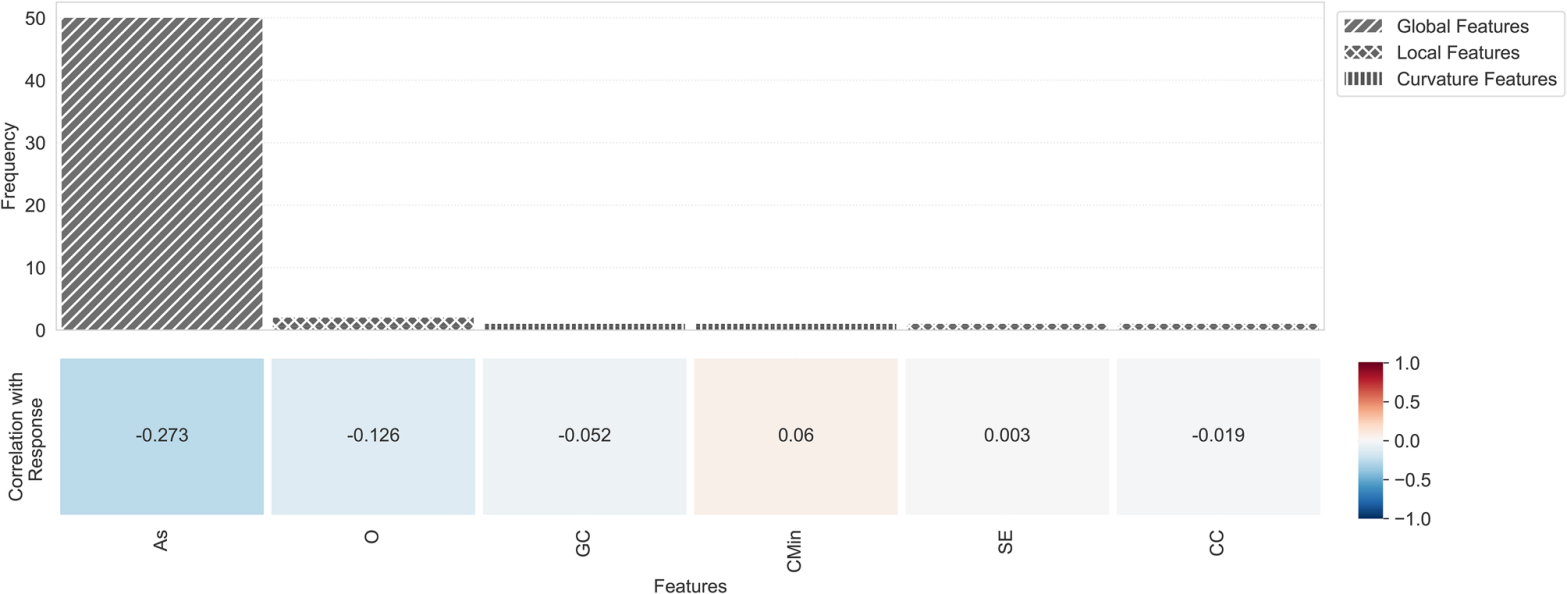
Top panel: the frequency of the selected features by the LogitNet model across 50 CV evaluations (5 outer folds, 10 repeats). Bottom panel: the Pearson correlation of the selected features with the response variable. For feature name, abbreviations, see Table 4

Figure 9 depicts feature importance based on the frequencies of features selected by the LogitNet model across all folds. Feature frequencies indicated that asphericity was the most prominent and common feature contributing to the model’s prediction.

**Fig. 10:**
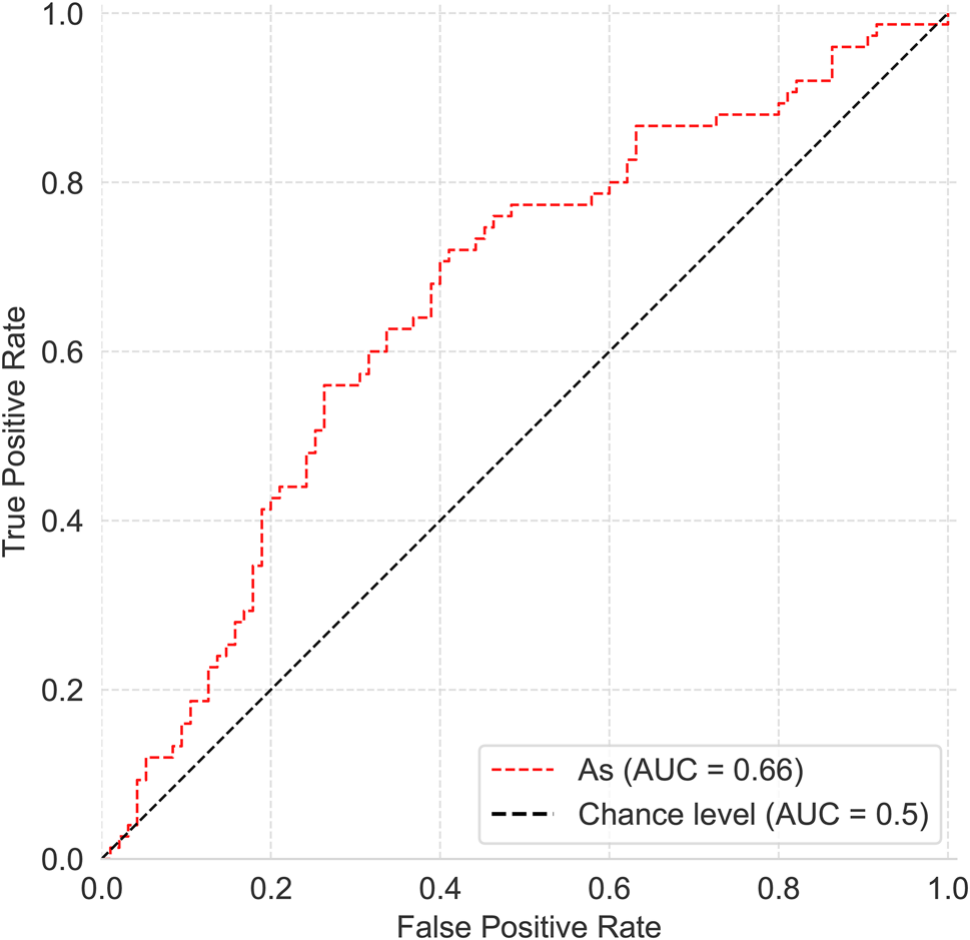
ROC curve with asphericity as the only feature

Furthermore, analyzing the As feature alone resulted in an AUC of 0.660, as shown in the ROC curve plot Fig. 10, suggesting minimal influence of other features on the LogiNet model.

### PCA experiment

We tested whether dimensionality reduction using Principal Component Analysis (PCA) Tipping and Bishop [38] would be beneficial prior to feature selection and classification. Before applying PCA, we standardized all features to have zero mean and unit variance using the StandardScaler from the SciKit-Learn library Pedregosa et al. [33]. PCA was applied to reduce dimensionality, retaining the components that explained at least 95% of the total variance.

The application of PCA did not lead to better classification outcomes Table 6. The LogitNet model, which utilized all 27 mesh features, achieved an AUC of 0.661 (SD = 0.066). In contrast, the LogitNet model built using 7 principal components through 44 folds and 8 components across the remaining 6 folds (which accounted for at least 95.0% of the total variance), resulted in a lower AUC of 0.604 (SD = 0.064). This suggests that, for this particular classification task, reducing dimensionality had no positive effect.

**Table 6:** PCA experiment results

Metric	Mean	SD
ROC-AUC	0.604	0.064
Balanced Accuracy	0.596	0.058
Sensitivity	0.622	0.106
Specificity	0.569	0.122

The mean and SD of ROC-AUC, balanced accuracy, sensitivity, and specificity from repeated cross-validation

## Discussion

In this study, we propose an innovative automated technique for quantifying the geometry of lateral ventricles. This method indicates asphericity as the most prominent feature in shunt surgery outcome prediction. Additionally, this pipeline is agnostic to both imaging modality and brain region, provided that 3D segmentations are available. Current international and Japanese guidelines highlight the critical role of radiology in diagnosing iNPH Relkin et al. [34]; Nakajima et al. [29]. Despite their relevance, these features are indicative rather than definitive, highlighting the limitations of current diagnostic markers Agerskov et al. [2]; Thavarajasingam et al. [37]. In contrast to traditional 2D imaging biomarkers, which primarily focus on linear indices, such as EI, CA, and BVR Relkin et al. [34]; Agerskov et al. [2]; Kockum et al. [19]; Nakajima et al. [29]; Thavarajasingam et al. [37], our method captures 3D shape properties that may deliver a wider range of insights into ventricular morphology.

From a conceptual perspective, our methodology bears similarities to the quantitative DESH assessment proposed by Yamada et al. [45]. However, the key difference in our approach is the utilization of a diverse array of features that encapsulate global, local, and curvature characteristics, in contrast to Yamada’s method, which relies on volume and volume ratios. Moreover, within the scope of our analysis, volume was not registered as an important feature, suggesting its limited capacity to capture the lateral ventricle intricacies associated with the reversibility of the iNPH.

Although not directly comparable due to the differing datasets employed, our result of AUC of 0.661 is favorable compared to prior studies that utilized the CA as a predictor for shunt surgery outcomes in patients with iNPH. For instance, Grahnke et al. [15] reported an AUC of 0.640, while Gao et al. [10] observed an AUC of 0.620. On the other hand, it is important to note that our findings did not reach the performance level of a multimodal approach that combined imaging features of DESH and CA, alongside the response to the tap test, which yielded an AUC of 0.720 Gao et al. [10].

Surprisingly, the results obtained from As as a standalone feature yielded an AUC of 0.660. This finding suggests that the contributions of other features to the performance of the LogiNet model were limited. Furthermore, this observation may explain why applying PCA for dimensionality reduction did not improve the results. The strength of our study lies in the utilization of multi-site, real-world data. Furthermore, the features employed in our analysis were derived from MRI segmentations rather than directly from MRI scans. The use of the cNeuro tool for segmenting MRI images offsets potential discrepancies or heterogeneity across scans from different vendors. On the other hand, there are a few limitations. Our pipeline’s ability to capture a wide range of features involves complex steps to convert segmentations into 3D meshes, with limited heuristics available for appropriate parameter selection. Also, we should generalize these results with caution, as our data was collected from a limited catchment area dominated by a single ethnicity. Furthermore, this methodological study aimed to characterize the new measures. A multifactorial analysis evaluating their prognostic value compared to or combined with other prognostic biomarkers is a topic for future work.

We suggest that future research explore how proposed features, especially asphericity, compare to traditional indices or whether combining these approaches could yield better results. Additionally, the potential of these features should be examined in a multimodal context, particularly when combined with biological or genetic markers. Also, a more complex analysis could consider the full range of curvatures and local values to identify specific regions of the ventricles that most significantly influence predictions. Furthermore, investigating the optimization of mesh parameters and proposing an optimization protocol would add significant value.

## Data Availability

The feature extraction pipeline is available online at (https://github.com/AP-environment/3D-Mesh-Feature-Extraction). Research data cannot be shared publicly due to the risk of violating patient privacy.
